# Spontaneous Cognition and Epistemic Agency in the Cognitive Niche

**DOI:** 10.3389/fpsyg.2018.00931

**Published:** 2018-06-08

**Authors:** Regina E. Fabry

**Affiliations:** Experimental Psychology and Cognitive Science, Department of Psychology, Justus Liebig University Giessen, Giessen, Germany

**Keywords:** spontaneous cognition, epistemic agency, mind wandering, depressive rumination, creative cognition, embodied cognition, embedded cognition, cognitive niche construction

## Abstract

According to Thomas Metzinger, many human cognitive processes in the waking state are spontaneous and are deprived of the experience of epistemic agency. He considers mind wandering as a paradigm example of our recurring loss of epistemic agency. I will enrich this view by extending the scope of the concept of epistemic agency to include cases of depressive rumination and creative cognition, which are additional types of spontaneous cognition. Like mind wandering, they are characterized by unique phenomenal and functional properties that give rise to varying degrees of epistemic agency. The main claim of this paper will be that the experience of being an epistemic agent within a certain time frame is a relational phenomenon that emerges from the organism’s capacity to interact with its cognitive niche. To explore this relation, I develop a new framework that integrates phenomenological considerations on epistemic agency with a functional account of the reciprocal coupling of the embodied organism with its cognitive niche. This account rests upon dynamical accounts of strong embodied and embedded cognition and recent work on cognitive niche construction. Importantly, epistemic agency and organism-niche coupling are gradual phenomena ranging from weak to strong realizations. The emerging framework will be employed to analyze mind wandering, depressive rumination, and creative cognition as well as their commonalities and differences. Mind wandering and depressive rumination are cases of weak epistemic agency and organism-niche coupling. However, there are also important phenomenological, functional, and neuronal differences. In contrast, creative cognition is a case of strong epistemic agency and organism-niche coupling. By providing a phenomenological and functional analysis of these distinct types of spontaneous cognition, we can gain a better understanding of the importance of organism-niche interaction for the realization of epistemic agency.

## Introduction

Mind wandering, depressive rumination, and creative cognition are types of spontaneous cognition (Christoff et al., 2016). They are ubiquitous phenomena that often contribute to our cognitive lives during wakefulness. Over the past few years, empirical research on spontaneous cognition has provided new insights into the phenomenological, functional, and neuronal properties of mind wandering (Mooneyham and Schooler, 2013; Schooler et al., 2014; Smallwood and Schooler, 2015; Konishi and Smallwood, 2016), depressive rumination (Koster et al., 2011; Christoff et al., 2016), and creative cognition (Dixon et al., 2014; Fox and Christoff, 2014; Christoff et al., 2016). What is lacking, however, is an overarching conceptual framework that can account for the commonalities and differences of these different types of spontaneous cognition. This paper is an attempt to help close this gap by developing a framework for the conceptually coherent and empirically plausible investigation of spontaneous cognition.

To this end, I will first describe and integrate the conceptual components that make important theoretical contributions to the emerging framework. In particular, I will suggest in Section “Toward a New Conceptual Framework for Investigating Spontaneous Cognition” that Metzinger’s (2013, 2015, 2017a,b) concepts of attentional and cognitive agency, originally applied to cases of mind wandering, can contribute to a better understanding of spontaneous cognition more generally construed. Furthermore, I will propose to approach spontaneous cognition from the perspective of strongly embodied and embedded cognition and cognitive niche construction. In the second part of the paper, I will explore mind wandering (see section “Mind Wandering and Weak Epistemic Agency in the Cognitive Niche”), depressive rumination (see section “Depressive Rumination and Weak Epistemic Agency in the Cognitive Niche”), and creative cognition (see section “Creative Cognition and Strong Epistemic Agency in the Cognitive Niche”) within this new framework. I will conclude by suggesting that attentional and cognitive agency, in conjunction with describing spontaneous cognition as a gradual phenomenon of organism-niche coupling, can lead to new theoretical insights into the phenomenological, functional, and neuronal signatures of mind wandering, depressive rumination, and creative cognition.

## Toward a New Conceptual Framework for Investigating Spontaneous Cognition

### Epistemic Agency

According to Metzinger (2013, 2015), mind wandering can be described as a transient loss of epistemic agency. Epistemic agency is realized by attentional agency and cognitive agency. Attentional agency is defined as “the ability to control one’s focus of attention” and cognitive agency refers to “the ability to control goal/task-related, deliberate thought” (Metzinger, 2013, p. 2). Attentional and cognitive agency are dispositional capacities that may or may not be phenomenological characteristics of cognitive processes within a certain temporal frame. Attentional agents phenomenally experience the ability to actively direct their attention toward objects, persons or states of affairs in the local environment that are relevant for the completion of cognitive tasks (e.g., arithmetic, reading for comprehension, writing a scientific paper). Cognitive agents have the phenomenal experience of being able to actively shape and modify a certain cognitive process that is often, but not always, directed toward a cognitive end (e.g., completing a calculation, comprehending a certain text, presenting a new scientific idea). As attentive and cognitive agents, we experience ourselves as “entities that actively construct and search for new epistemic relations to the world and to ourselves” (Metzinger, 2015, p. 274). On Metzinger’s construal, both attentive and cognitive agency qualify as cases of epistemic agency. Conceptually, attentional agency can occur without cognitive agency and can probably be experienced not only by humans, but also by other animals (Metzinger, 2015). However, attentional agency is necessary for the manifestation of cognitive agency in the case of human organisms (Metzinger, 2015, 2017b).

Epistemic agency is a gradual phenomenon ranging from weak to strong attentional and cognitive agency depending on the type of conscious cognition and the current phenomenological and functional profile of the human organism. In contrast to Metzinger’s conceptualization, I will argue that human organisms during wakefulness never fully lose epistemic agency. The reason is that human organisms need to maintain at least weak epistemic agency in order to be able to re-gain a robust sense of attentional and cognitive agency at some point. If attentional and cognitive agency were lost altogether in some cases, for example during mind wandering episodes, it would be difficult to describe the re-emergence of epistemic agency on a phenomenological level.

The degree of epistemic agency indicates the degree of *mental autonomy* for any given time frame (Metzinger, 2013, 2015, 2017b). Mental autonomy is defined as “the specific ability to control one’s own mental functions” (Metzinger, 2015, p. 276). ^1^ It is co-extensive with what has been called *meta-awareness* (Smallwood et al., 2007b; Schooler et al., 2011) and *meta-cognition* (Fox and Christoff, 2014) in the empirical literature on mind wandering. Like epistemic agency, mental autonomy is a gradual phenomenon (Metzinger, 2017a). It is further specified by the assumption that mentally autonomous human organisms have the ability to exert *veto control* over their current cognitive processes (Metzinger, 2013, 2015, 2017b). Veto control can be understood as the “intentional inhibition, suspension, or termination of an ongoing process” (Metzinger, 2013, p. 4). In sum, attentional and cognitive agency are cases of epistemic agency. Epistemic agency is a gradual phenomenon that indicates the degree of mental autonomy, and thus the manifestation of veto control, for any given time frame.

Metzinger’s (2013; 2015) conception of epistemic agency leaves room for the explicit assumption that the phenomenological and functional profile of human organisms within a certain time frame is defined in terms of their current relation to the local environment. This is suggested by the idea that mind wandering, which will be classified as a case of weak epistemic agency (see section “Mind Wandering and Weak Epistemic Agency in the Cognitive Niche”), is depicted as a process that is partly defined by the organism’s “lack of sensitivity to the external situational context” (Metzinger, 2015, p. 274). This assumption is corroborated by the explanatory dimensions that have been employed to systematize mind wandering and other types of spontaneous cognition.

### Explanatory Dimensions

Empirical researchers investigating mind wandering and other types of spontaneous cognition have specified their target phenomena along several dimensions. First, cognitive processes have been described as being either task-related or task-unrelated (Smallwood, 2011; Broadway et al., 2015; Metzinger, 2015; Smallwood and Schooler, 2015; Christoff et al., 2016; Irving, 2016). The idea is that mind wandering is unrelated to a cognitive task within a well-defined experimental context (e.g., reading for comprehension). The explanatory dimension of task-relatedness/unrelatedness is problematic, because human organisms are bound to complete several cognitive tasks at any given time (Metzinger, 2017b). Even if a primary cognitive task is defined by the design of a certain study, this will not rule out the possibility that participants’ cognitive processes cannot be exhaustively assessed relative to this task.

Second, cognitive processes have been systematized as being either goal-directed or goal-undirected (Mooneyham and Schooler, 2013; Christoff et al., 2016; Irving, 2016). This explanatory dimension faces similar difficulties like the task-relatedness/unrelatedness dimension, because it remains unclear whether or not human organisms pursue one and only one epistemic goal within a given time frame.

Third, it has been argued that mind wandering and other types of spontaneous cognition can be specified along the dimension of stimulus-dependence/-independence of cognitive processes (Schooler et al., 2011; Broadway et al., 2015; Smallwood and Schooler, 2015; Christoff et al., 2016; Konishi and Smallwood, 2016). The problem with this dimension is that it is difficult to specify the class of stimuli that are relevant for an explanation of mind wandering and other types of spontaneous cognition. The reason is that cognitive processes are always causally dependent upon a plethora of exteroceptive and interoceptive stimuli (Metzinger, 2017b).

Finally, it has been suggested that perceptual coupling/decoupling is an explanatory dimension that helps specify the relation between internally or externally directed cognitive processes (Schooler et al., 2011; Smallwood, 2011; Broadway et al., 2015; Smallwood and Schooler, 2015; Konishi and Smallwood, 2016; Sanders et al., 2017). The distinction between internally and externally directed cognition is not without its difficulties (Dixon et al., 2014), because it induces an internalism/externalism dualism that cannot do conceptual justice to the delicate interplay of human organisms and their local environment (see sections “Embodied Cognition and Organism-Niche Interaction” and “Organism-Niche Interaction and Epistemic Agency”). However, the general idea that cognitive processes are realized by varying degrees of coupling between organisms and their local environment gives rise to a promising explanatory dimension that could contribute to a nuanced characterization of different types of spontaneous cognition. The coupling/decoupling dimension can be further specified by taking the embodiedness and embeddedness of cognitive processes into account.

### Embodied Cognition and Organism-Niche Interaction

Accounts of embodied cognition subscribe to the idea that embodied action contributes to cognitive processes (Rowlands, 1999; Gallagher, 2005; Menary, 2007; Clark, 2008; Anderson et al., 2012; Chemero, 2013). Proponents of a strong embodiment thesis are committed to the view that the embodied interaction with the local environment plays an indispensable functional role in at least some cognitive processes (Menary, 2015a; Fabry, 2018). There is ample empirical evidence in support of the strong embodiment thesis in domains that are relevant for theoretical considerations on various types of cognition. For example, results from eye-tracking and behavioral studies attest to the indispensability of the bodily manipulation of symbols for calculation (Dinehart and Manfra, 2013; Hartmann, 2015; Mock et al., 2016), of eye movements for reading (Gilchrist et al., 1997; Rayner, 1998, 2009), and of hand and arm movements for writing (Teulings et al., 1983; Dounskaia et al., 2000; Phillips et al., 2009). One important implication of the strong embodied cognition thesis is that the decrease of embodied interaction patterns leads to a decrease of the functional realization of the associated cognitive processes. This will become relevant for the forthcoming considerations on mind wandering and depressive rumination (see sections “Mind Wandering and Weak Epistemic Agency in the Cognitive Niche” and “Depressive Rumination and Weak Epistemic Agency in the Cognitive Niche”).

A subscription to the strong embodiment thesis is connected to the commitment to a strong variant of the embedded cognition thesis. According to this thesis, at least some cognitive processes are realized by the integration of cerebral, extra-cerebral bodily, and environmental components (Menary, 2015a; Fabry, 2018). If correct, the strong embedded cognition thesis gives rise to the view that plausible and coherent accounts of at least some cognitive processes – cases of spontaneous cognition included – should take the embodied interaction with the local environment, in which human organisms are embedded, into serious consideration.^2^ In the human case (and in the case of other animals as well), the local environment can be specified by the notion of the *cognitive niche*.

The cognitive niche is the result of the *trans*-generational realization, cultural inheritance, and modification of artifacts, practices, institutions, and learning opportunities in the local environment (Sterelny, 2003, 2012; Clark, 2008; Stotz, 2010; Kendal, 2011; MacKinnon and Fuentes, 2012; Bertolotti and Magnani, 2016). Continuous with other animals, human organisms have constructed their niche over multiple generations so as to render it optimally, or near-optimally tied to their cognitive processing needs. If it is correct to say that at least some cognitive processes – including spontaneous cognitive processes – are embedded in the cognitive niche in a strong sense, then the entire “brain-body-niche nexus” becomes highly relevant for accounts of spontaneous cognition (Menary, 2015b, p. 3).

An advantage of this perspective is that it helps specify the coupling/de-coupling dimension. The notion of coupling, as it is widely used in the empirical literature on mind wandering, can be enriched by taking the technical term of *reciprocal coupling* into account, which has been employed in dynamical systems theory (Beer, 1995, 2000; van Gelder, 1998; Barandiaran and Moreno, 2006; Schöner, 2008). The term of reciprocal causal coupling and the conceptual and mathematical framework provided by dynamical systems theory has inspired and enriched philosophical research on strongly embodied and embedded cognition (Clark, 1997; Chemero, 2000; Menary, 2007, 2015a). Dynamical systems theory provides us with conceptual and mathematical tools that specify the concurrent dynamical reciprocal interaction of embodied organisms and their local environment (i.e., the cognitive niche):

*Because an agent [A] and its environment [E] are in constant interaction, A and E are coupled non-autonomous dynamical systems. This coupling can be represented with a sensory function S from environmental state variables to agent parameters and a motor function M from agent state variables to environmental parameters* (Beer, 1995, p. 130).

Coupled dynamical systems are mathematically represented as moving through their state space across time. In this way, reciprocal coupling provides us with a better understanding of the explanatory dimension of coupling/decoupling. Strongly coupled dynamical systems are characterized by a tight reciprocal relationship of the organism and its cognitive niche. By contrast, in cases of weak coupling, the reciprocal interaction of the organism and the cognitive niche is diminished and leads to profound changes to sensory and motor functions, which represent a decrease of the coupling relation. Reciprocal coupling is not an all-or-nothing phenomenon but allows for matters of degree with weak coupling and strong coupling as ends of a continuum of different configurations of organism-niche interaction. In Section “Epistemic Agency,” I have suggested that human organisms never fully lose epistemic agency but maintain at least a weak degree of attentional and cognitive agency. Correspondingly, human organisms are never fully de-coupled from their cognitive niche, but remain at least minimally coupled to it. This is because human organisms need to be coupled to their cognitive niche in order to maintain their physical integrity.

### Organism-Niche Interaction and Epistemic Agency

In what follows, I rely on the idea that at least some cognitive processes relevant for considerations on spontaneous cognition are strongly embodied and embedded and that the technical term of reciprocal coupling provides us with conceptual resources to specify the relation between organisms and their cognitive niche within a certain time frame. My proposal is to relate a personal-level phenomenological analysis of epistemic agency to a sub-personal functional analysis of reciprocal coupling of the embodied organism and the cognitive niche. As I will show in the next sections, this approach to spontaneous cognition can provide us with a better understanding of the commonalities and differences of mind wandering, depressive rumination, and creative cognition.

The phenomenological term of epistemic agency can be enriched by showing that it corresponds to the functional-level analysis of reciprocal coupling. In particular, on a phenomenological, personal level of analysis, epistemic agency is the experience of being an embodied organism interacting with the cognitive niche. On a functional, sub-personal level of analysis, epistemic agency is realized by a strong reciprocal coupling relation of the embodied organism and the cognitive niche. Furthermore, epistemic agency is a gradual phenomenon on a phenomenological, personal level. It ranges from weak to strong experiences of interacting with the cognitive niche. This corresponds to the functional, sub-personal level insight that reciprocal coupling is a gradual phenomenon ranging from weakly to strongly embodied interactions of human organisms with their cognitive niche.

Recently, Metzinger (2017a, p. 7) has suggested that cognitive agency can be understood “as an abstract mental simulation of embodied actions, first executed using the physical, non-neural body.” By contrast, I assume that embodied actions, represented by the motor function linking the organism to the cognitive niche, play an indispensable role in the functional manifestation of cognitive agency. As we shall see in the next two sections, mind wandering and depressive rumination are cases of weak cognitive agency (and thus weak epistemic agency). The reason is that these cognitive processes are depleted of robust patterns of embodied interaction with the cognitive niche. In contrast to mind wandering and depressive rumination, creative cognition is associated with strong epistemic agency and a strong reciprocal coupling relation between the embodied organism and the cognitive niche (see section “Creative Cognition and Strong Epistemic Agency in the Cognitive Niche”). The upshot of this view is that embodied action itself – and not its simulation – is of crucial importance for determining the degree of epistemic agency across different types of spontaneous cognition.

At first, glance, this strategy to describe the relationship between human organisms and the cognitive niche in terms of reciprocal coupling bears interesting similarities to *wild systems theory* (Jordan and Vinson, 2012; Jordan, 2013; Jordan and Day, 2015; Jordan et al., 2017).^3^ This theory “conceptualizes bodies as *self-sustaining, multi-scale embodiments* of the phylogenetic, cultural, and ontogenetic *contexts* in which they emerged and in which they sustain themselves” (Jordan and Vinson, 2012, p. 7; italics in original). Wild systems theory and the present account share the idea that the relationship between organisms and their niche is important for considerations on the cognitive lives of human organisms. Upon further scrutiny, however, there are at least two non-trivial differences that set my account apart from wild systems theory. First, my account and wild systems theory differ in their meta-theoretical commitments. This difference can be made clear with reference to van Gelder’s (1998) distinction between the knowledge hypothesis and the nature hypothesis that proponents of dynamical systems theory could endorse: “The *nature* hypothesis is a claim about the nature of cognitive agents themselves; it specifies what they are (i.e., dynamical systems). The *knowledge* hypothesis is a claim about cognitive science: namely, that we can and should *understand* cognition dynamically” (van Gelder, 1998, p. 619; italics in original). Wild systems theory proposes a nature hypothesis that is connected to a knowledge hypothesis about empirical and theoretical research on *wild systems*. Wild systems theory proposes an “*ontology* of ubiquitous, multi-scale relationality” that seamlessly links embodied organisms and their current physical and socio-cultural contexts (Jordan et al., 2017, p. 2; emphasis added). For this reason, wild systems theory “renders properties that had been historically associated with the subjective, such as phenomenology, value, and meaning […] *constitutive* of what organisms *are*” (Jordan and Day, 2015, p. 19; emphasis added). Furthermore, one of the goals of wild systems theory is to develop “a metaphysics of meaning” about the relationship of organisms and their environment (Jordan and Vinson, 2012, p. 19). By contrast, my account offers a knowledge hypothesis that is interested in the question how spontaneous cognition can be understood and how considerations on different levels of analysis relate to each other. At present, my account does not offer a nature hypothesis and is agnostic about its potential ontological and metaphysical implications, without denying that it will be important to think about these implications in future research.

Second, the different meta-theoretical commitments of wild systems theory and my account lead to theoretical differences, which concern the relevance to deny that there is an *epistemic gap* that separates human organisms and their environment (Jordan and Day, 2015). Given the characterization of the relationship between embodiment and context by the proponents of wild systems theory, “embodied contexts are necessarily about the contexts they embody, there is no *epistemic gap* between an organism and its environment” (Jordan and Vinson, 2012, p. 9; italics in original). For current purposes I am agnostic to the question whether or not there is an epistemic gap in a robust ontological or metaphysical sense. However, I do think that the notion of an epistemic gap can be applied to my previous considerations on epistemic agency and the coupling of human organisms to their niche. On a phenomenological level, it is possible to ask whether the phenomenal experience of an epistemic gap is characteristic of mind wandering or depressive rumination, for example. On a functional level, I submit that the very principle of reciprocal coupling presupposes that the coupled systems are functionally distinct. Otherwise it would be hard to see why they should be coupled – either weakly or strongly – in the first place under a functional description. In this restricted functional sense, human organisms and their niche would be separated by an epistemic gap, but it would be in virtue of this gap – and not despite of it – that organisms and their niche can be strongly coupled in principle. In sum, while I think that wild systems theory offers intriguing nature and knowledge hypotheses about the relationship of human organisms and their local environment, my account has different meta-theoretical and theoretical commitments. The remainder of this paper provides detailed considerations on the knowledge hypothesis. The core idea is that we can arrive at a better understanding of mind wandering, depressive rumination, and creative cognition by establishing a link between phenomenological-level descriptions of epistemic agency and functional-level assessments of the reciprocal coupling of human organisms and the cognitive niche.

## Mind Wandering and Weak Epistemic Agency in the Cognitive Niche

Research in the cognitive sciences was long based on the implicit assumption that our cognitive endeavors are pervasively characterized by attentional and cognitive agency. However, recent advances in the systematic research on mind wandering suggest that our cognitive processes are not always strongly connected to the cognitive niche. Rather, mind wandering appears to be an important part of our cognitive lives (Metzinger, 2013; Mooneyham and Schooler, 2013; Schooler et al., 2014; Konishi and Smallwood, 2016). In an attempt to avoid the conceptual problems of the explanatory dimensions identified in Section “Explanatory Dimensions,” I define mind wandering as the transient phenomenal and functional disentanglement of human organisms and their cognitive niche.

During mind wandering episodes, attention is no longer actively directed toward specific objects, persons, or states of affairs in the cognitive niche that contribute to the completion of the current primary cognitive task, as it is defined in an experimental context (Mooneyham and Schooler, 2013; Schooler et al., 2014; Broadway et al., 2015; Smallwood and Schooler, 2015; Sanders et al., 2017). This is the case for both tuning out and zoning out, which are two types of mind wandering that have been identified in the literature (Schooler et al., 2004, 2011; Smallwood et al., 2007b; Dixon et al., 2014; Metzinger, 2015; Smallwood and Schooler, 2015). In cases of *tuning out*, human organisms can become aware that they are mind wandering and are able to control their train of thought at least to some degree. By contrast, in cases of *zoning out*, human organisms are unaware that they are mind wandering, but they phenomenally experience the contents of their mind wandering episodes. In other words, “they are experientially conscious of whatever topic has grabbed their attention, while at the same time lacking metaconsciousness of the fact that they are zoning out” (Schooler et al., 2004, p. 203). Given that the majority of empirical and conceptual investigations has focused on cases of zoning out, I will restrict my considerations to these cases in this paper. However, I do not deny that tuning out is an interesting phenomenon in its own right that deserves a more detailed treatment in future research.

In cases of zoning out, mind wandering is characterized by “unguided attention” (Irving, 2016, p. 563). This means that constraints on the direction of attention are relaxed, which gives rise to phenomenal experiences that are at least partly independent from the current situation in the cognitive niche (Dixon et al., 2014; Schooler et al., 2014; Broadway et al., 2015; Konishi and Smallwood, 2016). In other words, mind wandering episodes show “a lack of sensitivity to the situational context” (Metzinger, 2015, p. 274). They can be sustained for an extended period of time, because they are accompanied by “a temporary failure of meta-awareness” (Smallwood et al., 2007b, p. 527). They are only terminated when meta-awareness (and thus mental autonomy) is regained, i.e., when the organism becomes aware that it was not aware of the current situation in the cognitive niche, because it was mind wandering.

Mind wandering is associated with neuronal activation patterns in cortical areas contributing to the *default mode network*, especially in the posterior cingulate cortex and the medial prefrontal cortex (Fox and Christoff, 2014; Broadway et al., 2015; Smallwood and Schooler, 2015). Other areas include the lateral prefrontal cortex, the medial parietal cortex, the lateral parietal cortex, and parts of the temporal lobe (Schooler et al., 2011; Dixon et al., 2014; Christoff et al., 2016). Neuronal activations in cortical areas contributing to what is now known as the default mode network were initially interpreted as realizers of resting states correlated with baseline conditions in neuroimaging experiments (Christoff et al., 2016). It was not until the late 1990s that a systematic and invariant cortical network was identified that was activated during alleged resting states (Shulman et al., 1997; Raichle et al., 2001). Recently, it has been reported that activations in the default mode network are frequently anti-correlated with the dorsal attention network (Christoff et al., 2016). This network is comprised by the intraparietal sulcus and the superior parietal lobe, the frontal eye field, and motor areas located in the middle temporal lobe. It is associated with the direction of attention toward the cognitive niche and with the co-ordination of sensori-motor processes.

One of the most frequent ways to empirically investigate mind wandering has been to employ a reading task paradigm (Schooler et al., 2004; Smallwood et al., 2008; Smallwood, 2011; Uzzaman and Joordens, 2011; Sanders et al., 2017). In these studies, participants are asked to read a text for comprehension. Mind wandering episodes are identified based on participants’ self-reports, which are either probe-caught (Smallwood et al., 2008; Uzzaman and Joordens, 2011; Sousa et al., 2013; Broadway et al., 2015), self-caught (Sanders et al., 2017), or caught by a combination of both (Reichle et al., 2010). Toward the end of the experiments, participants are asked to complete a reading comprehension test. On a behavioral level, the results indicate that mind wandering is reliably and consistently associated with poor reading comprehension (Schooler et al., 2004, 2014; Franklin et al., 2011; Smallwood and Schooler, 2015). The onset of mind wandering episodes has a negative impact on reading comprehension at word, sentence, and text levels.

Several eye-tracking studies suggest that mind wandering episodes are associated with eye movement patterns that are distinct from those identified for episodes of attentive reading (Reichle et al., 2010; Smilek et al., 2010; Uzzaman and Joordens, 2011). In comparison to attentive reading, mind wandering episodes are associated with fewer fixations, longer fixation durations, and less regressions. Furthermore, these studies suggest that eye movement patterns during mind wandering episodes do not indicate any (unconscious or conscious) sensitivity to the lexical properties of the reading material. It is generally assumed that the frequency and predictability of words in a semantic and syntactic context have a strong influence on the duration of fixations and the probability of word skipping (Rayner, 1998; Drieghe et al., 2004; Kliegl et al., 2004). Highly frequent and predictable words in a given context are likely to be either skipped or fixated for a shorter period of time in comparison to words of average or low frequency and predictability, based on statistical estimates and corpus analyses. The frequency and predictability effects appear to be absent during mind wandering episodes (Schooler et al., 2014). This is consistent with findings from an EEG study indicating that event-related potentials associated with the reading process at lower levels, P1 and N1, are less pronounced during mind wandering episodes in comparison to attentive reading episodes (Broadway et al., 2015). In sum, studies on the impact of mind wandering on reading comprehension have made important contributions to the empirical investigation of the phenomenal and functional properties of mind wandering episodes.

On a phenomenological level, mind wandering episodes (in cases of zoning out) are characterized by minimal epistemic agency. The reason is that human organisms are neither in a position to actively control their focus of attention, nor are they actively and deliberately selecting and maintaining the targets of their cognitive processes (Metzinger, 2013, 2015). This indicates that mind wandering human organisms are temporarily unable to realize that they are mind wandering. Furthermore, their capacity to intentionally inhibit their cognitive processes is transiently diminished (Metzinger, 2013, 2015).

On a functional level, mind wandering episodes are cases of weak reciprocal coupling of the embodied organism and its cognitive niche. Weak reciprocal coupling is indicated by the decrease of fixations and fixation durations reported in the above-mentioned eye-tracking experiments. If eye movements are a type of embodied action and if the frequency and duration of eye movements decrease as a function of mind wandering, then the embodied action that establishes the reciprocal coupling relation between the organism and the cognitive niche is weak. On this construal, attention becomes an inherent phenomenon of reciprocal coupling of the embodied organism and the cognitive niche: In terms of dynamical systems theory, attention is functionally expressed by the relation between the sensory and motor functions. If the organism-niche system is only weakly coupled, this gives rise to weak attentional agency as it is approached on a phenomenological level. Toward the end of a mind wandering episode, the degree of attentional and cognitive agency increases. Functionally, this corresponds to an increase of organism-niche coupling.

## Depressive Rumination and Weak Epistemic Agency in the Cognitive Niche

According to Christoff et al. (2016), depressive rumination is characterized by changes to the phenomenological, functional, and neuronal profile in comparison to non-pathological types of spontaneous cognition. It is a pervasive symptom of major depressive disorder, which belongs diagnostically to the cluster of depressive disorders. In addition to depressive rumination, other symptoms of major depressive disorder include the experience of depressed mood, a loss of interest in previously enjoyable activities, social withdrawal, fatigue, and stupor (DSM-5 American Psychiatric Association, 2013). On a phenomenological level, major depressive disorder is characterized by a pervasive experience of loss. What is lost is the possibility to interact meaningfully with persons and objects in the cognitive niche. This experience of loss is often associated with “a sense of estrangement” and a “feeling of isolation” (Ratcliffe, 2015, p. 71). During major depressive episodes, “the possibility of interpersonal connection” has vanished (Ratcliffe, 2015, p. 218). At the same time, the social world is often experienced as threatening, malevolent, or ignorant.

In addition, major depressive disorder is associated with changes to the experience of one’s own body, which includes fatigue, numbness, and a deceleration of movements or an inability to move (e.g., in speech and locomotion). Furthermore, “experiences of heaviness, exhaustion, and lack of vitality” are often reported by individuals suffering from a major depressive episode (Ratcliffe, 2015, p. 76). In many cases, the experienced impossibility to bodily interact with the cognitive niche and to engage socially with other persons gives rise to perceptual, cognitive, and affective withdrawal. Withdrawal is often connected to depressive rumination in a feedback-loop.

In rumination, attention is directed toward repetitive, monothematic, and negatively valenced cognitive processes that are disentangled from the current situation in the cognitive niche (Nolen-Hoeksema et al., 2008; Dixon et al., 2014; Christoff et al., 2016; Irving, 2016). In many cases, attention is focused on “self-referring negative information” (Koster et al., 2011, p. 140). Examples include repetitive cognitive processes directed toward one’s own distress, loss, failures, and deficits. These cognitive processes prevent the emergence of phenomenal experiences of being an active cognizer who is meaningfully embedded in the cognitive niche.

Importantly, “excessive stability” is a key property of ruminative cognitive processes (Christoff et al., 2016, p. 8; see also Irving, 2016). The stability and repetitiveness of rumination is closely linked to the all-encompassing feeling of stasis and hopelessness. Phenomenologically, “the world of depression is bereft of even the *possibility* of change” (Ratcliffe, 2015, p. 65; italics in original). Changes are brought about by embodied interactions with the cognitive niche. If embodied interactions are experienced as impossible, due to bodily feelings of numbness, weakness, or fatigue, the likelihood of the co-occurrence of withdrawal and rumination increases. Withdrawal leads to rumination, because the human organism directs its attention away from the cognitive niche and toward its own deficits and conflicts. At the same time, the stability of negatively valenced ruminative cognitive processes reinforces the tendency toward perceptual, cognitive, and affective withdrawal. This feedback-loop linking withdrawal and rumination contributes to the pervasive and concurrent phenomenal experience of loss and impossibility.

On a neuronal level, Christoff et al. (2016) indicate that depressive rumination is associated with significant activation patterns in the default mode network. In addition, the fronto-parietal control network shows increased functional connectivity with the default mode network and decreased functional connectivity with the dorsal attention network. Areas contributing to the fronto-parietal control network include the dorsolateral prefrontal cortex and the anterior inferior parietal lobe. This pattern of functional connectivity is entirely consistent with the phenomenological idea that rumination and withdrawal from the cognitive niche are closely linked.

On a phenomenological level, depressive rumination is another case of minimal epistemic agency. However, as we will see shortly, it has a markedly different phenomenological, functional, and neuronal profile than mind wandering. During depressive rumination, human organisms experience minimal attentional agency, because they are transiently incapable to direct their attention away from their distressful, negatively valenced cognitive processes. This is in line with the idea that depressive rumination can be labeled as an attentional control deficit, which manifests itself as “a difficulty to exercise attentional control in response to negative thoughts” (Koster et al., 2011, p. 139). Irving (2016) has also suggested that depressive rumination is associated with weak attentional agency. On his view, “[w]hen someone ruminates, her attention needn’t be guided toward information that seems relevant to any of her goals” (Irving, 2016, pp. 566–567). If my account of depressive rumination, and its relation to other symptoms of major depressive disorder, is largely correct, it will become clear that we have to go one step further in our characterization of weak attentional agency. In cases of rumination, human organisms are transiently incapable of directing their attention toward any goals, because major depressive disorder is characterized by the *absence* of goals, which is closely connected to feelings of hopelessness and stasis.

Minimal attentional agency is accompanied by minimal cognitive agency, because depressive individuals have transitorily lost control over the targets of their cognitive processes, which includes that they cannot terminate them at will. Put differently, “individuals with depression may want to stop themselves from ruminating but are often unable to do so” (Christoff et al., 2016, p. 8). This suggests that the weakness of epistemic agency is available to meta-cognitive processes. However, this meta-cognitive insight on its own is not sufficient for an increase of epistemic agency. If correct, the availability of meta-cognitive insight about the currently weak phenomenal manifestation of epistemic agency lends support to the idea that epistemic agency is never fully lost, but continues to be weakly experienced. If epistemic agency were fully lost, it would be hard to see how the meta-cognitive insight into the current level of attentional and cognitive agency could be experienced. Minimal epistemic agency, in combination with the meta-cognitive insight, often leads to a reinforcement of the other symptoms of major depressive disorder, because rumination continues to contribute to the withdrawal-rumination feedback-loop.

Functionally, depressive rumination is a case of weak organism-niche coupling. The reason is that both sensory and motor functions linking the organism and the cognitive niche fall below their optimal values. Relating this functional analysis to the phenomenology of depressive rumination and other symptoms of major depressive disorder, we can see that both sensory and motor functions are compromised. Perceptual, cognitive, and affective withdrawal and depressive rumination are associated with sub-optimal sensory functions. At the same time, bodily feelings of fatigue, numbness, and a deceleration or impossibility of movements are associated with sub-optimal motor functions. Depressive rumination, and the larger symptom cluster of major depressive disorder, can lend support to the idea that the coupling relation of human organisms and their cognitive niche is realized by sensory and motor components, which influence each other in a reciprocal fashion. If the reciprocal coupling is significantly weakened, as it appears to be the case in major depressive disorder, this can have severe, often harmful consequences.

Metzinger (2015) indicates that depressive rumination is an example of mind wandering. At first glance, this seems justified, given that both mind wandering and depressive rumination are characterized by minimal epistemic agency that can be described in terms of weak organism-niche coupling on a functional level. Upon further consideration of the phenomenological signature of rumination, however, the target and the affective valence of both types of spontaneous cognition are markedly different. This is in line with the following suggestion: “when we consider the dynamics of thought, mind-wandering and rumination seem antithetical: although thoughts during mind-wandering are free to ‘move hither and thither,’ thoughts during rumination tend to remain fixed on a single theme or topic” (Christoff et al., 2016, p. 2). In other words, the phenomenal experience of mind wandering displays a non-trivial degree of flexibility and variability. By contrast, in depressive rumination, the target of cognitive processes is inflexible and invariable, just as the entire phenomenal experience of major depressive disorder is interspersed with the feeling of stasis and the loss of changeability.

For this reason, there are also important differences between depressive rumination and the negative impact of mind wandering episodes on general mood (Mooneyham and Schooler, 2013; Schooler et al., 2014). For example, Smallwood et al. (2007a) have argued that there is a link between the frequency of dysphoria associated with mind wandering episodes on the one hand and the onset of depressive disorder on the other hand. Against this view, I propose that experiences of dysphoria that are reported to be related to the frequency of mind wandering episodes are distinct from the phenomenal experiences of depressive rumination and other manifestations of major depressive disorder.

On a functional level, mind wandering episodes and rumination are different, because they have different temporal dynamics. Mind wandering episodes, as they are individuated by empirical research, operate at the order of seconds and minutes. By contrast, episodes of depressive rumination are usually more persistent and can also last for hours and entire days without interjected stages of stronger organism-niche coupling. Put differently, depressive rumination is specified by an alternation of weak and strong reciprocal coupling that operates at a longer time scale in comparison to mind wandering. This consideration is in line with Metzinger’s (2017b) request that we need to find and establish temporal criteria for the individuation of episodes of mind wandering – and depressive rumination.

Finally, mind wandering and depressive rumination should also be distinguishable on a neuronal level. Both types of spontaneous cognition are associated with significant activation patterns in the default mode network. However, we should expect to find important differences in the functional connectivity of the default mode network with areas contributing to the dorsal attention network, the fronto-parietal control network, and other cortical areas. Currently, neuroscientific studies on the neuro-functional realization of depressive rumination are sparse. However, based on the consideration that major depressive disorder is also associated with the dysfunction of sub-cortical structures, such as the amygdala and the hypothalamus (Barrett and Simmons, 2015; Badcock et al., 2017), it is likely that mind wandering and depressive rumination show important neuro-functional differences across both cortical and sub-cortical areas.

In sum, depressive rumination is a case of weak epistemic agency on a phenomenological level and of weak organism-niche coupling on a functional level. The discussion in this section shows that mind wandering and depressive rumination are distinct types of spontaneous cognition.

## Creative Cognition and Strong Epistemic Agency in the Cognitive Niche

Recently, the empirical and theoretical investigation of spontaneous cognition has been extended to include cases of creative cognition (Dixon et al., 2014; Fox and Christoff, 2014; Christoff et al., 2016). In this section, I argue that creative cognition is a type of spontaneous cognition that is markedly different from mind wandering and depressive rumination. This is because it is at the opposite end of the continuum of spontaneous cognition in terms of the manifested degree of epistemic agency and organism-niche coupling. In general, creativity can be defined as “the ability to come up with ideas or artifacts that are new, surprising, and valuable” (Boden, 2004, p. 1). In this sense, creativity is a dispositional component of our cognitive lives that can manifest itself in processes of creative cognition. The initial working definition of creativity already suggests a distinction between creative cognitive processes and creative products (Fox and Christoff, 2014). Whether or not a cognitive process counts as creative is dependent upon the appreciation of the properties of the creative product. In the existing literature on creativity, there have been different proposals about the properties that a creative product ought to have: it is supposed to be novel, original, and unique (Boden, 2004; Carruthers, 2011; Beaty et al., 2016), valuable (Boden, 2004; Carruthers, 2011; Wiggins et al., 2015; Stokes and Paul, 2016), useful (Carruthers, 2011; Fink and Benedek, 2013; Beaty et al., 2016), surprising (Boden, 2004; Carruthers, 2011), or a combination of these properties. The scope of these attributions suggests that the definition of creative cognition as well as the appreciation of creative products are dependent upon the norms and conventions that govern the interaction of human organisms with their cognitive niche. Put differently, “creativity does not happen inside people’s heads, but in the interaction between a person’s thought and a sociocultural context” which is provided by the cognitive niche (Csikszentmihalyi, 2013, p. 23).

Creative cognition can occur in many domains of the arts, humanities and sciences (Vygotsky, 2004; Wiggins et al., 2015), ranging from visual art, music, and neuroscience to film, literature, engineering, biology, and philosophy. It is often characterized by the recombination of ideas, artifacts, or symbolic representations that have already been part of the cognitive niche before the beginning of the creative process. In Vygotsky’s words, “[i]t is this ability to combine elements to produce a structure, to combine the old in new ways that is the basis of creativity” (Vygotsky, 2004, p. 12).

Creative cognition is construed as a process that integrates the *generation* and the *evaluation* of creative products (Boden, 2004; Carruthers, 2011; Beaty et al., 2016; Christoff et al., 2016). The integration of the generation and evaluation of creative products is also indicated by phenomenological self-reports by artists, scientists, and inventors (Csikszentmihalyi, 2013).

Several fMRI studies suggest that creative cognitive processes are associated with the interaction of areas contributing to the default mode network and of areas contributing to the fronto-parietal control network (Shah et al., 2013; Beaty et al., 2015, 2016; Liu et al., 2015; Christoff et al., 2016). The suggestion is that creative generation is associated with activations contributing to the default mode network. Creative evaluation is supposed to be associated with activations in functionally connected areas that are part of the default mode network and the fronto-parietal control network.

Research has begun to develop neuroimaging paradigms that allow experimenters to combine a quest for ecological validity with the reduction of errors in the spatial resolution of blood oxygen-level dependent (BOLD) signal measures. For example, two fMRI studies on creative hand-writing, where participants are asked to write short continuations of literary narrative texts, show that areas contributing to the default mode network and to the fronto-parietal control network interact with each other in significant ways (Shah et al., 2013; Erhard et al., 2014). Furthermore, areas in the motor cortex and visual cortex and bilateral occipito-temporal areas contribute to the entire cerebral process underlying creative hand-writing (Shah et al., 2013). Activations in these areas are associated with the sensori-motor processes and lower-level processing and production routines underlying reading and hand-writing (Dehaene et al., 2010; Price and Devlin, 2011; Purcell et al., 2011, 2017; Kersey and James, 2013; DeMarco et al., 2017).

Consistent with the studies by Shah et al. (2013) and Erhard et al. (2014). Liu et al. (2015) report an fMRI study which investigated the neural correlates of the creative type-writing of poems. In this study, participants are required to create poems on-line while using a keyboard and a computer screen showing the written material. The important finding of this study are significant neuronal activation patterns in the middle prefrontal cortex, which contributes to the default mode network, and in the dorsolateral prefrontal cortex as well as areas in the parietal cortex, which contribute to the fronto-parietal control network. These areas are functionally connected and interact with each other throughout the creative writing process. Liu et al. (2015) also find that areas in the dorsolateral prefrontal cortex are functionally connected to sensori-motor areas.

Another illustrative example of neuroimaging research on creative cognition is a study on jazz improvisation in expert musicians (Limb and Braun, 2008). Musical improvisation is defined “as the immediate, on-line improvisation of novel melodic, harmonic, and rhythmic musical elements within a relevant musical context” (Limb and Braun, 2008, p. 1). The findings of this study indicate that creative musical improvisation is associated with activations in the medial prefrontal cortex, which contributes to the default mode network. At the same time, the dorsolateral prefrontal cortex, which contributes to the fronto-parietal control network, was deactivated in the improvisation condition. This suggests that creative generation and creative evaluation are probably not two consecutive stages, but are dynamically integrated. Part of the reason might be that musical improvisation is a creative task that is realized under strong time constraints, which put demands on the flexibility and speed of the creative cognitive process. Indeed, commenting on the study by Limb and Braun (2008); Fox and Christoff (2014) suggest that musical improvisation is a case of creative cognition “wherein the two states of creative thinking (i.e., generation and evaluation) are condensed into one, and metacognitive evaluation accompanies spontaneous ideation quasi-simultaneously” (Fox and Christoff, 2014, p. 311). This leaves room for the possibility that a clear-cut, principled distinction between creative generation and evaluation might be an artifact of the particular block design that is employed by Shah et al. (2013), Erhard et al. (2014), and other studies on creative cognition. Another interesting finding of the study by Limb and Braun (2008) is that jazz improvisation is associated with activations in ventral and dorsal lateral motor areas, the supplementary motor area, and in portions of the primary motor cortex, which suggests that associated sensori-motor processes make important contributions to the creative process.

In contrast to mind wandering and depressive rumination, creative cognition is a case of strong epistemic agency.^4^ Creative human organisms phenomenally experience attentional agency, because they are able to voluntarily direct and re-direct their attention toward elements in the cognitive niche that are currently relevant for the creative process. Furthermore, they can also direct their attention toward their embodied interaction with artifacts and tools in the cognitive niche, for example, the way they are holding their hands during writing or playing the piano. Importantly, attentional agents can also direct their attention away from their embodied action patterns and elements in the cognitive niche in order to facilitate the creative process.

Creative human organisms are also cognitive agents, because they are able to control the target and the temporal unfolding of their cognitive processes. They are apt to phenomenally experience aesthetic qualities (e.g., the beauty of a metaphor, the sonority of a triad) or the re-combination of artifacts, symbolic representations, or tonalities in the flux of the creative process. Cognitive agency is about the phenomenal experience of being an active creator of a new idea, artifact, symbolic representation, or musical piece. Importantly, cognitive agency is established by the experience of actively engaging with elements in the cognitive niche (e.g., a clavier, pen and paper, keyboard and CPU) through concurrent embodied action.

This view is consistent with descriptions of creative *flow* experiences. While in flow, “we are aware only of what is relevant here and now,” which is the “result of intense concentration on the present” (Csikszentmihalyi, 2013, p. 112). At first glance, flow, which is often associated with “self-forgetfulness,” might be at odds with the postulation of strong epistemic agency (op. cit., p. 113). However, this only poses a problem if a strong sense of self is necessary for epistemic agency. I suggest that it is theoretically possible that epistemic agency can be realized without the phenomenal experience of a strong sense of self. One example that might lend support to this general possibility is the class of phenomenal experiences associated with mindfulness meditation (Metzinger, 2015). It could be the case that the experience of flow in cases of creative cognition can at least sometimes become an important phenomenological component of epistemic agency.

In the recent literature on spontaneous cognition, it has been suggested that mind wandering and creative cognition are similar, perhaps even co-emergent. First, Metzinger (2013, p. 4) suggests that cases of creative cognition could be “interesting situations where human beings quickly alternate between mind wandering and short episodes of M-autonomy (i.e., mental autonomy).” I do not intend to deny that there can be cases in which M-autonomy diminishes and re-emerges at a considerably short time scale. However, I would like to suggest that at least in a large number of cases, creative cognitive processes are characterized by strong cognitive agency, and thus by mental autonomy, from beginning to end. This is because creative cognizers are able to voluntarily direct their attention and to control their unfolding cognitive process.

Second, Smallwood and Schooler (2015, p. 507) suggest that mind wandering and creative cognition are strikingly similar: “A fundamental similarity exists between the creative experience and the self-generated thoughts that arise during mind wandering: both are illustrative of experiences people generate that are discrepant from the current or dominant psychological interpretations of the task environment.” If my perspective on creative cognition is largely correct, then creative cognitive processes are much more strongly coupled to the current environment, i.e., the cognitive niche, than indicated by Smallwood and Schooler.

Third, it is argued that mind wandering is beneficial for creative cognition, because it is an opportunity for *creative incubation* (Mooneyham and Schooler, 2013; Schooler et al., 2014). However, creative incubation, as it is currently studied empirically, is restricted to a very narrow domain, namely to divergent thinking, which is often investigated by the *unusual use task* paradigm. In this paradigm, participants are asked to generate unusual ways of using a certain familiar object. There are three options: first, we could grant that performing the unusual use task with an interval of incubation is a case of creative cognition, which renders the link between mind wandering and creative cognition much closer than initially suggested. Second, we could propose that the completion of the unusual use task is not a case of creative cognition as it is defined here, but of divergent problem solving. This would leave the phenomenological, functional, and neuronal distinctions of mind wandering and creative cognition put forward in this section unaffected. Finally, we could suggest that the performance in the unusual use task requires both divergent problem solving and creative cognition. At least in some cases, mind wandering could make positive contributions to creative problem solving, but it would not be an indispensable condition for task performance. This proposal would not be in conflict with my clear-cut distinction of mind wandering and creative cognition. At the same time, it would be flexible enough to allow for cases in which creative processes contribute to the completion of cognitive tasks in other domains, such as problem solving. Given the current empirical evidence on creative incubation effects, which is limited to the unusual use task paradigm, and experimental results from studies on creative writing and musical improvisation reported above, the second and third options appear to be more tenable at the moment than the first option.

Creative cognition, as it has been conceptualized in this section, is a case of strong epistemic agency. Strong epistemic agency on a phenomenological level corresponds to strong reciprocal organism-niche coupling on a functional level. During creative processes, human organisms are in constant interaction with elements in the cognitive niche. They shape, sculpt, and recombine these elements through embodied interaction. At the same time, the concurrently manipulated elements in the cognitive niche feed back into the organism’s ways of perceiving its current material, socio-cultural situation. This description of strong reciprocal coupling is a linguistic expression of the sensory and motor functions that are expressed mathematically by dynamical systems theory.

The idea that the strong coupling relation between the creative human organism and its cognitive niche is realized by embodied actions is supported by the indispensable contribution of hand and arm movements and of neuronal activations in sensori-motor areas to creative writing and musical improvisation in the studies reported by Limb and Braun (2008), Shah et al. (2013), and Erhard et al. (2014). Without these embodied action patterns, which are spanning the brain and the rest of the body, creative processes such as creative writing and musical improvisation would be very different, if not completely impossible.

In relation to mind wandering and depressive rumination, creative cognition is at the opposite end of the epistemic agency-coupling continuum of spontaneous cognition. Creative cognition is about the close entanglement with the cognitive niche through active and exploratory cognitive processes. By contrast, mind wandering is about the temporary *dis*entanglement of concurrent cognitive processes from the cognitive niche. Creative cognition is also different from depressive rumination, because it is characterized by the flexible integration of the embodied interaction with the cognitive niche into cognitive processes. By contrast, depressive rumination is closely connected to perceptual, cognitive, and affective withdrawal from the cognitive niche. Furthermore, creative cognition is characterized by the flexible flow of active cognitive processes that contribute to the emergence of creative products. As we have already seen in Section “Depressive Rumination and Weak Epistemic Agency in the Cognitive Niche,” depressive rumination is a manifestation of repetitive, stable, invariant, and negatively valenced self-referential cognitive processes. These contrasts between creative cognition on the one hand and mind wandering and depressive rumination on the other hand highlight the importance of the embodied interaction with the cognitive niche for productive, constructive, and malleable cognitive processes in the here and now.

## Concluding Remarks

The purpose of this paper has been to develop a new framework for empirical and philosophical research on spontaneous cognition. I have suggested that the integration of Metzinger’s (2013, 2015, 2017a,b) work on epistemic agency with research on strongly embodied and embedded cognition and cognitive niche construction leads to a new and productive way to think about mind wandering, depressive rumination, and creative cognition. The resulting new framework operates on phenomenological, functional, and neuronal levels of analysis. According to the considerations put forward in this paper, mind wandering and depressive rumination are cases of weak epistemic agency and weak organism-niche coupling. By contrast, creative cognition is characterized by strong epistemic agency and a strong coupling relation between the embodied organism and its cognitive niche. Throughout the discussion and informed by empirical research, I have pointed out that there are important commonalities and differences of these distinct types of spontaneous cognition. Future research in philosophy and the cognitive sciences is clearly needed to specify the conditions of mind wandering, depressive rumination, and creative cognition. For the time being, I hope to have shown that the framework developed in this paper can enrich current research on spontaneous cognition by providing a new way to think about epistemic agency and the relation of human organisms to their cognitive niche.

## Author Contributions

The author confirms being the sole contributor of this work and approved it for publication.

## Conflict of Interest Statement

The author declares that the research was conducted in the absence of any commercial or financial relationships that could be construed as a potential conflict of interest.

## Footnotes

1 Metzinger’s (2013, 2015) concept of *epistemic agency* is different from the concept of *epistemic agency* as it is employed in virtue epistemology. According to Elgin’s (2013) neo-Kantian *epistemic imperative*, “an epistemic agent should believe only considerations that she could advocate and accept as a legislating member of a realm of epistemic ends” within a certain epistemic community (p. 144). Epistemic agency in this sense includes the ability for belief justification and belief revision in interlocution with other members of an epistemic community. Epistemic agency in Metzinger’s sense, which is characterized by mental autonomy, can sometimes be conducive to epistemic agency in Elgin’s sense, but this is not a conceptual or empirical requirement. This is suggested by Metzinger’s view that “mental autonomy can be a form of rational self-control, which is based on reasons, beliefs, and conceptual thought, but it does not have to be” (Metzinger, 2013, p. 4).

2 The purpose of this paper is to develop a positive proposal for the investigation of different types of spontaneous cognition. This proposal is committed to strong theses about the embodiment and embeddedness of at least some cognitive processes and therefore belongs to a class of theoretical accounts of embodied, embedded, extended, and enactive (4E cognition). It is beyond the scope of the paper to survey the theoretical landscape of research on 4E cognition. For an overview of different accounts of 4E cognition and their theoretical commitments, see Newen et al. (in press).

3 Thanks to a reviewer for bringing this issue to my attention.

4 This classification of creative cognition is theoretically independent from the empirical question whether or not creative generation and evaluation are clearly distinguishable stages of creative cognitive processes.
